# An application of the augmented synthetic control method within a target trial framework: the case of the soda tax policy in California

**DOI:** 10.1186/s12889-025-22526-5

**Published:** 2025-04-11

**Authors:** Fan Zhao, Risha Gidwani, May C. Wang, Liwei Chen, Roch A. Nianogo

**Affiliations:** 1 Department of Epidemiology, Fielding School of Public Health, University of California, Los Angeles (UCLA), Los Angeles, CA USA; 2 Department of Health Policy and Management, Fielding School of Public Health, University of California, Los Angeles (UCLA), Los Angeles, CA USA; 3 RAND Corporation, Santa Monica, CA USA; 4 Department of Community Health Science, Fielding School of Public Health, University of California, Los Angeles (UCLA), Los Angeles, CA USA; 5 California Center for Population Research (CCPR), Los Angeles, CA USA

**Keywords:** SSB tax, Augmented synthetic control, Obesity, California, Trial emulation framework

## Abstract

**Background:**

Sugar-sweetened beverage (SSB) consumption is associated with increased obesity risk. One microeconomic intervention approach that has been studied is the increase of the cost of SSBs through SSB taxes. This study aims to apply the augmented synthetic control method (ASCM) within a target trial framework to estimate the impact of a 1-cent-per-ounce SSB tax on obesity prevalence in California.

**Methods:**

We used 2012–2020 data from the California Health Interview Survey (CHIS)’s AskCHIS Neighborhood Edition (AskCHIS NE) and the American Community Survey (ACS). The outcome of interest was obesity prevalence at the city level for people aged 18 and older. The estimated effect of the policy was calculated as the difference between the observed outcome in each soda tax city in the post-policy period and the predicted outcome in the synthetic controls in the post-policy period. The causal estimand of interest was the average treatment effect among the treated (ATT). We adjusted for sex, age, employment status, education, race/ethnicity, marital status, poverty, household median income, population size, and percentage of people who took public transportation to work.

**Results:**

Relative to not implementing a soda tax, the mean difference in obesity prevalence three years after the implementation of a soda tax was -5.5 (95%CI -34.9 to 21.1) percentage points (pp) in Berkeley, -1.7 (95%CI, -11.3 to 6.8) pp in Albany, -1.0 (95%CI, -6.5 to 4.3) pp in Oakland, and 2.6 (-11.0 to 16.8) pp in San Francisco. Overall, the mean difference in obesity prevalence was -1.4 (95%CI, -9.2 to 5.7) pp.

**Conclusions:**

In this study, we illustrated the use of the augmented synthetic control methodology within a target trial framework with group-level longitudinal data. Our estimates of the impact of SSB tax policy on the obesity prevalence in California were imprecise.

**Supplementary Information:**

The online version contains supplementary material available at 10.1186/s12889-025-22526-5.

## Background

Over a span of just 20 years (2000–2020), the prevalence of adult obesity in the US increased by nearly 40%, from 30.5% to 41.9% [1]. Obesity contributes to preventable, non-communicable diseases including heart disease, stroke, type 2 diabetes, and certain types of cancer [1]. In 2019, the annual medical cost of obesity was $173 billion in the US [1]. Medical costs for adults with obesity are $1,861 higher than people with healthy weight [1]. Consumption of foods high in added sugar increases the risk of obesity [2].

Sugar sweetened beverages (SSBs) are the largest source of added sugars in the American diet, comprising 36% of all added sugar consumption [3]. SSBs are drinks with added caloric sweeteners, including non-diet soft drinks/sodas, flavored juice drinks, sports drinks, sweetened tea, coffee drinks, energy drinks, and electrolyte replacement drinks [4]. Americans consume the most SSBs globally at 216 L per person per year, nearly twice as much as the second country on the list [5]. Fifty six percent of American adults aged 20 years and over failed to meet the Dietary Guidelines for Americans recommendation of limiting added sugar intake to less than 10% of daily calories in 2017 [6].

Given the high levels of soda consumption in the US, reducing soda consumption may be an effective strategy for reducing obesity prevalence [7]. Borrowing from the field of microeconomics, one strategy for reducing soda consumption is to increase the price of soda by imposing a SSB excise tax. In the US, a SSB excise tax is based on the volume of the beverage, e.g. $0.01 per liter. SSB tax is collected from manufacturers (rather than consumers), resulting in higher shelf prices that consumers *see*when making decisions [8]. Regular sales taxes, in contrast, are collected at check-out and usually implemented for revenue generation rather than for changing consumption behavior, and tend to have less of an effect on soda consumption and obesity rates [9, 10].

Four cities in California – Berkeley, Albany, Oakland, and San Francisco – passed a 1-cent-per-ounce SSB tax, with Berkeley first implementing such a law in 2015 [11, 12]. Taxed SSBs include soda, sports drinks, energy drinks, and sweetened ice teas but exclude milk-based beverages, meal replacement drink, diet sodas, fruit juice, and alcohol [13]. Studies have shown that SSB sales as well as consumption decreased within one year of tax implementation [14–16]. Such decreases were sustained after 2 or 3 years too [17, 18]. SSB tax may also be an equitable public policy. Jones-Smith et al. found that while lower income populations paid a higher percentage of their income in beverage taxes, there was no difference in absolute spending on beverage taxes per capita, and that there was a sizable net transfer of funds towards programs targeting lower income populations. [19] Thus, when considering both population-level taxes paid and sufficiently targeted allocations of tax revenues, a sweetened beverage tax may have characteristics of an equitable public policy. Due to the fact that current soda tax does not account for inflation, price effects on soda consumption may change over time, needing longer-term evaluation [14].

In this study, we investigated the impact of the SSB tax on adult obesity prevalence in California. We implemented the augmented synthetic control method (ASCM) within a target trial framework [20–22].

## Methods

### Study design

Our study follows the target trial framework to estimate observational analogues to intention-to-treat effects by using observational data to emulate a hypothetical randomized trial [23–25]. The intervention would consist of randomly assigning some cites to the intervention arm (they will adopt a 1-cent-per-ounce SSB tax.) while the control will continue with the status quo. These cities will be followed for 3 years (since this is the smallest number of years an individual city has been followed, e.g. San Francisco in our case; this will ensure the results are comparable across treated cities). An intent-to-treat analysis will then be conducted by contrasting the obesity prevalence in cities assigned to the intervention group to those assigned to the control group. This will allow for estimation of single effect by city. However, the results could then be aggregated across the individual cities [26]. Further details on the protocol of the target trial are described below and in Supplement Table 1.

### Data sources

*SSB tax information*was collected from city government websites [7]. Yearly *city-level socio-demographic*data were obtained from the Census Bureau’s American Community Survey (ACS) 2012–2020. All Californian cities were included. (Supplement Fig. 1, Supplement Text 2) ACS is an annual nation-wide survey that collects social, economic, housing and demographic information on the US population [27].*City-level obesity prevalence estimates*among people aged 18 and older in Californian cities were obtained from the California Health Interview Survey (CHIS)’s AskCHIS Neighborhood Edition (AskCHIS NE). CHIS is a multistage cross-sectional survey of California households conducted every year since 2011 by the University of California, Los Angeles (UCLA) Center for Health Policy Research [28]. AskCHIS NE provides city-level model-based estimates of key health behaviors and conditions including city-level obesity prevalence [29]. More details on the methodology for obtaining small area can be found in the Supplemental Text 1.

### Target trial protocol

We have adopted the target trial framework as it evaluates eligibility criteria and defines interventions as well as outcomes in a way that is compatible with a randomized control trial. This is particularly appealing given that randomized trials for a SSB tax policy are not practical to implement. Furthermore, the goal of target trial emulation is to ensure that observational analyses preserve desirable features of randomized trials, e.g., randomization and time zero, to draw causal inference [30]. In this design the protocols are explicitly specified (including defining the causal estimand) in a transparent manner so that the quality of evidence can be assessed [26].

#### Eligibility criteria and exposures

The exposure variable was the implementation of the SSB tax. Cities in California that have implemented the SSB tax policy (Berkeley, Albany, Oakland and San Francisco) were considered treated cities. A previous study showed that people are willing to travel and make purchases within 12 miles when the price is less in neighboring cities [31]. Spillover can happen for instance when individuals who live outside the cities where SSB tax are implemented, occasionally shop in cities where SSB tax are implemented and thus be exposed to the tax when shopping there. As such, we limited control cities to those that were beyond 12 miles from SSB tax cities given the possibility of spillover effects.

A total of 121 eligible control cities were included. (More details on their identities can be found in the Supplement Fig. 3, Supplement Text 4) The SSB tax was implemented at different times across cities. The tax took effect on 1/1/2015 in Berkeley, 4/1/2017 in Albany, 7/1/2017 in Oakland and 1/1/2018 in San Francisco [11, 12, 32]. (Supplement Fig. 1) We considered the period 2012–2014 as the pre-tax period for Berkeley; the period 2012–2016 the pre-tax period for Albany and Oakland; and the period 2012–2017 the pre-tax period for San Francisco.

#### Treatment strategy

The treatment strategy or intervention was a city-level 1-cent-per-ounce SSB tax. Taxed SSBs include soda, sports drinks, energy drinks, and sweetened ice teas but exclude milk-based beverages, meal replacement drink, diet sodas, fruit juice, and alcohol [13].

#### Treatment assignment

**Fig. 1:**
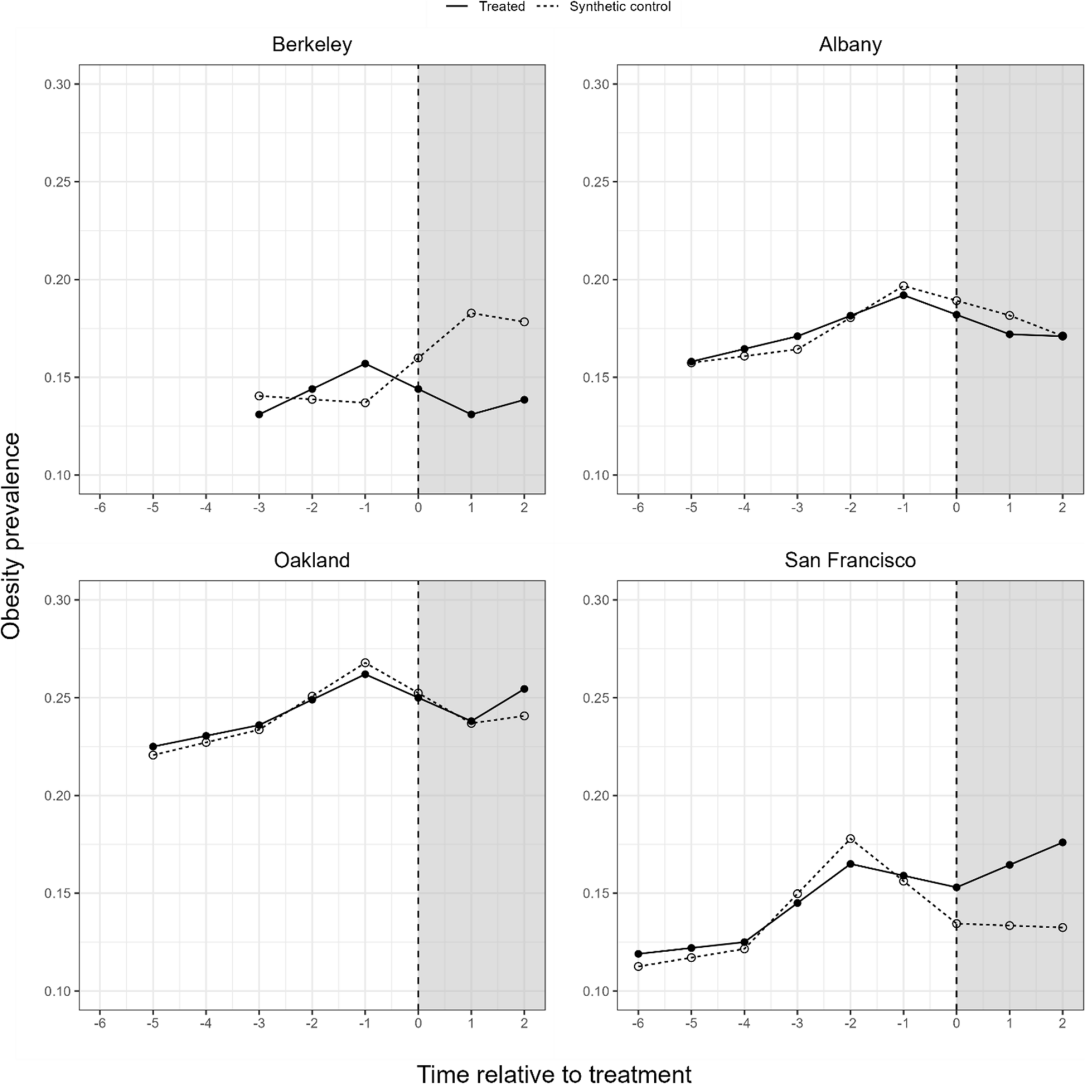
Obesity prevalence among people who were 18 years and older from 2012 to 2020 in treated cities (i.e., implemented the tax policy) and their corresponding synthetic controls (i.e., counterfactual, average obesity prevalence in treated cities had they not been treated). Shaded areas represent the periods after the soda tax was implemented. The soda tax was implemented on 1/1/2015 in Berkeley, 4/1/2017 in Albany, 7/1/2017 in Oakland, and 1/1/2018 in San Francisco. Solid circles and lines represented obesity prevalence in SSB tax cities, hollow circles and dashed lines represented obesity prevalence in synthetic control cities

The time at which the SSB tax took effect in the treated cities (Berkeley, Albany, Oakland and San Francisco) were considered time zero and the cities were aligned on time since the SSB tax policy has been enacted [11, 12, 32]. (Fig. 1) Eligible controls were used to create a weighted (synthetic) control city for each treated city.

#### Outcome assessment

Our outcome was the obesity prevalence among people aged 18 and older in each city. AskCHIS NE provided city-level model-based small area estimates of obesity prevalence [33].

#### Causal contrast

The intention-to-treat effect was obtained by contrasting the obesity prevalence in cities that have implemented the SSB tax to that in the corresponding weighted (synthetic) control. The synthetic control was a weighted average of controls. This is the average treatment effect among the treated (ATT) and the ATT in each treated unit can then be aggregated to obtain the overall effect [34, 35]. ATT was estimated because we were only interested in the average treatment effect of those treated [26, 35].

### Statistical analysis

To estimate the extent to which implementing the SSB tax was associated with a change in obesity prevalence, we employed the augmented synthetic control method (ASCM). ASCM is a novel analytical method that allows for the creation of “synthetic controls” from a pool of *donor*controls which may be individually inadequate but weighted to generate a single counterfactual city that resembles a treated city on most characteristics at the exception of treatment. ASCM uses a ridge regression regularization and applies a set of weight to control units such that the trajectory of outcome of the weighted average of the available controls is similar to the trajectory of the outcome of the treated unit in the pre-treatment periods [34, 36, 37]. Ridge ASCM penalizes distance from sparse nonnegative SCM weights [34]. The estimated effect of the policy is calculated as the difference between the observed outcome in each soda tax city in the post-policy period and the predicted outcome in the synthetic controls in the post-policy period. This is the average treatment effect among the treated (ATT) and the ATT in each treated unit can then be aggregated to obtain the overall effect [34, 35]. ATT was estimated because we were interested in the effect of the SSB tax policy on obesity prevalence in cities that adopted the SSB tax policy. ATT was estimated by contrasting the average obesity prevalence in cities that have adopted the SSB tax policy, $\text{E}\left[\text{Y}|\text{A}=1\right]$, to that in cities that have adopted the SSB tax policy had these cities not adopted the SSB tax policy, i.e., the counterfactual cities (also the synthetic control), $\text{E}\left[{\text{Y}}^{\text{A}=0}|\text{A}=1\right]$.

Applying this concept to time-series data and adopting the notation by Xu [38], if Y_1_ is the postintervention outcome for the treated unit, and Y_0_ the postintervention outcome for untreated or control units, then the difference Y_1_ − Y_0_W is equal to the ATT. W is a weight estimated by the ASCM to minimize the pre-treatment difference between the treated group and the control group and Y_0_W represents the synthetic control, that is, a weighted average of controls. Essentially, synthetic control represents a counterfactual, that is, what would have happened to the obesity prevalence outcome in the city that adopted the SSB tax policy in the absence of the SSB tax policy. Confidence intervals were estimated based on the conformal inference approach of Chernozhukov et al. and were computed through permutation tests. [34, 39] More technical details are provided in the Supplement Text 2 and can also be found from the reference [34]. The ASCM was implemented in R via the *augsynth*package version 0.2.0 [40].

We adjusted for sex (% males), age (%18 years and older), employment status (% unemployed), education (% bachelor’s degree or higher), race/ethnicity (% non-White), marital status (% now married), poverty (% below poverty), household median income (log-transformed for normality), population size (log-transformed for normality), and percentage of people who took public transportation to work [27]. (Supplement Fig. 2).

### Imputation

City-level obesity prevalence data were only available in 2012, 2014, 2016, 2018 and 2020. Additionally, covariates data were not available for some cities in some years. Therefore, we imputed missing values using univariate time series imputation that employs time dependencies for other years using the R package *imputeTS*version 3.3 [41]. (See Supplement Text 3 and Supplement Fig. 4 for additional details) Data analysis was implemented in R software version 4.3.0 [42].

### Sensitivity analysis

Berkeley passed the SSB tax in 11/2014, Albany, Oakland, San Francisco passed the SSB tax in 11/2016 [11, 13, 43]. We conducted a sensitivity analysis where we took 1 year since pass-through of a tax as the washout period. We considered the period 2012–2015 as the pre-tax period for Berkeley; the period 2012–2017 as the post-tax period for Albany, Oakland, and San Francisco. We also implemented SCM as sensitivity analysis.

We additionally computed predicted mean squared errors (PMSE) to evaluate the pre-treatment fit of the main model because good pre-treatment fit was important when using ASCM [34]. To assess the results’ sensitivity to model specification, we also fit models that included different predictors, and computed PMSE of the different modeling choices [44, 45].

## Results

### Baseline characteristics

Table 1 shows the city characteristics in Berkeley, Albany, Oakland, San Francisco, and their respective synthetic control cities in the pre-soda tax period. The percentage of population who were unemployed was 8.7% and 6.9% for Berkely and synthetic Berkeley; 4.6% and 6.2% for Albany and synthetic Albany; 9.3% and 6.5% for Oakland and synthetic Oakland; 6.5% and 5.8% for San Francisco and synthetic San Francisco. The percentage of population who were Non-White was 72.8% in Oakland and 50.8% in synthetic Oakland; 59.7% in San Francisco and 51.7% and synthetic San Francisco; 50.9% in Albany and 51.7% in synthetic Albany; 44.6% in Berkeley and 50.5% in synthetic Berkeley. More than 20% of the population took public transportation to work in SSB tax cities. It was 22.0% in Berkeley, 23.9% in Albany, 20.5% in Oakland and 33.9% in San Francisco.

**Table 1:** City characteristics in pre-soda tax periods in California

Characteristic	Soda tax cities and corresponding synthetic controls								All control cities
	Berkeley	Synthetic Berkeley	Albany	Synthetic Albany	Oakland	Synthetic Oakland	San Francisco	Synthetic San Francisco	
Males, %	48.6(1.2)	48.1(1.9)	48.6(0.2)	48.3(1.9)	48.9(0.7)	48.3(2.0)	50.9(0.1)	48.3(1.9)	49.4(1.3)
Married, %	33.1(0.0)	50.2(0.9)	58.3 (1.4)	50.3(1.0)	38.0(0.6)	50.3(0.9)	39.1(1.1)	50.3(1.0)	47.1(5.3)
Unemployed, %	8.7(0.1)	6.9(0.5)	4.6(0.3)	6.2(0.6)	9.3(3.2)	6.5(0.6)	5.8(1.6)	6.2(0.6)	8.2(2.8)
Non-Whites, %	44.6(0.3)	50.5(22.4)	50.9(1.5)	51.7(21.6)	72.8(0.8)	50.5(22.1)	59.1(0.5)	51.7(21.6)	63.6(19.4)
Bachelor’s degree or higher, %	70.2(2.2)	52.8(11.1)	73.0(1.3)	54.9(10.9)	39.6(1.0)	54.3(10.5)	55.2(2.0)	54.9(10.9)	31.0(15.9)
Aged 18 years and older, %	87.3(0.1)	82.3(1.2)	73.5(0.5)	82.2(0.8)	79.4(0.7)	82.3(1.1)	86.6(0.0)	82.2(0.8)	75.7(3.8)
Taking public transportation to work, %	22.0(1.9)	15.9(3.8)	23.9(1.3)	15.9(3.7)	20.5(1.8)	15.9(3.8)	33.9(0.8)	15.9(3.7)	3.5(3.3)
Log median income, $	11.1(0.1)	11.3(0.1)	11.3(0.1)	11.4(0.1)	10.9(0.1)	11.3(0.1)	11.4(0.2)	11.4(0.1)	11.1(0.3)
Log population size	11.7(0.02)	11.3(0.2)	9.9(0.02)	11.3(0.2)	13.0(0.02)	11.3(0.2)	13.7(0.02)	11.3(0.2)	11.7(0.6)
Below poverty, %	14.0(0.5)	8.3(1.4)	10.7(0.4)	8.2(1.2)	19.7(1.6)	8.4(1.3)	12.2(1.4)	8.2(1.2)	13.5(5.8)

Mean and SD for all variables. Pre-soda tax periods were 2012–2014 for Berkley, 2012–2016 for Albany and Oakland, and 2012–2017 for San Francisco

In the pre-SSB tax years, mean obesity prevalence was 14.4% in Berkeley vs 16.3% in synthetic Berkeley. Mean obesity prevalence was 17.3% in Albany vs 17.4% in synthetic Albany; 24.1% in Oakland vs 17.4% in synthetic Oakland; and 13.9% in San Francisco vs 17.8% in synthetic San Francisco. (Supplemental Table 2) The fit of the pre-SSB tax period was good for the four SSB tax cities. (Fig. 1).

### Change in obesity prevalence

**Fig. 2:**
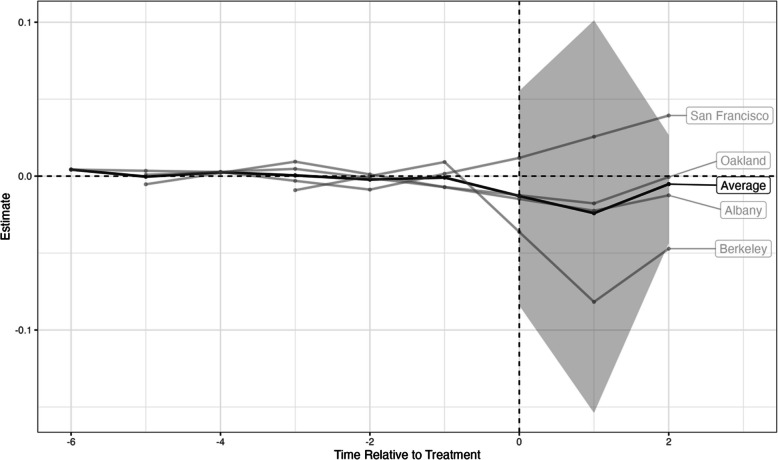
Impact of the soda tax policy on obesity prevalence in California cities estimated using the augmented synthetic control method. This impact corresponds to an average treatment effect on the treated (ATT)

Relative to not implementing a SSB tax, the mean difference in obesity prevalence three years after the SSB tax was -5.5 (95%CI -34.9 to 21.1) percentage points (pp) in Berkeley, -1.7 (95%CI, -11.3 to 6.8) pp in Albany, -1.0 (95%CI, -6.5 to 4.3) pp in Oakland, and 2.6 (-11.0 to 16.8) pp in San Francisco. Overall, the mean difference in obesity prevalence was -1.4 (95%CI, -9.2 to 5.7) pp. (Table 2, Fig. 2).

**Table 2:** Estimated obesity prevalence difference in percentage points after implementation of the soda tax

City, year of policy implementation	Adjusted obesity prevalence difference, %
Albany, 2017	-1.7 (-11.3, 6.8)
Berkeley, 2015	-5.5 (-34.9, 21.1)
Oakland, 2017	-1.0 (-6.5, 4.3)
San Francisco, 2018	2.6 (-11.0, 16.8)
Overall	-1.4 (-9.2, 5.7)

Model adjusted for age, sex, employment status, race/ethnicity, education, median log(income), marital status, log(population) size, and percentage of people taking public transportation to work. Mean and 95%CI for all estimates

### Sensitivity analysis

When we considered one year wash-out period after SSB tax pass-through, the results were similar. (Supplement Table 1) The pre-SSB tax period was short and pre-treatment fit was not as good when using SCM. (Supplement Fig. 5) PMSEs were the same (1.47) with different model specification. (Supplement Table 5.)

## Discussion

In this study, we illustrated the use of the augmented synthetic control methodology within a target trial framework with group-level longitudinal data. Our estimates of the impact of SSB tax policy on the obesity prevalence in California were imprecise.

Obesity is among important policy issues that local governments are increasingly inclined to address. Consuming SSBs increases overall energy intake, provides little to no nutritional value and may even encourage further energy intake because liquid foods have lower satiety than solid foods [46–48]. The population impact of a SSB tax intervention on reducing SSB consumption and consequently obesity risk is dependent on the magnitude of the response of consumers to increased SSB prices. Philadelphia experienced significantly great increases in taxed beverage prices and significantly large declines in volume of taxed beverages sold within one year of 1.5-cent-per-ounce SSB tax [49]. The reduction in taxed beverage volume sales was sustained 2 years after SSB tax implementation, and volume sales of nontaxed beverage concentrates increased on average [17]. In Oakland, roughly 60% of the tax was passed on to consumers in the form of higher prices within one year of SSB tax and here was a slight decrease in the volume of SSBs purchased per shopping trip [50].

While previous studies have found that soda purchase and consumption reduced after implementation of SSB tax, the evidence on the extent to which changes in SSB prices may impact weight outcomes is mixed [14, 31, 51–53]. Fletcher et al. estimated the impact of 2-cent-per-12-oz and 1-cent-per-12-oz soda tax increase in two states and found that there were no changes in Body Mass Index (BMI), overweight prevalence or obesity prevalence. [54] A 2010 study examined associations between existing SSB sales taxes ranging from 1.5% to 2.3%, and weight outcomes among children and adolescents and found little to no effect after 17 years of follow up [55]. Using US national birth certificate data, Jackson et.al found that SSB taxes were associated with a 41.4% decreased risk of gestational diabetes mellitus (GDM), a 7.9% reduction in weight-gain-for-gestational-age z-score, and decreased risk of infants born small-for-gestational-age [56]. Flynn used the Youth Risk Behavioral Surveillance System (YRBS) and found that Philadelphia high school students reduced consumption by over a soda per week for four full years following the tax and there were BMI reductions of 1.3% for students across Philadelphia, Oakland, and San Francisco [57, 58].

The possible explanations for the limited effect of SSB tax on weight changes observed in our study may be that the current SSB tax is not high enough to affect energy balance. A study of adults found an increase of one percentage point in the state SSB tax rate leads to a decrease in BMI of 0.003 points [59]. Modeling studies found that a 20% tax fully passed on to consumers resulted in a decrease of obesity prevalence by 3% over 10 years [60, 61]. In contrast, the SSB tax we evaluated here is a 1-cent-per-ounce tax. In California, the average price of sugary drinks is almost $0.059/ounce; a 1-cent-per-ounce tax would therefore raise prices by 16.9%, which is less than the 20% increased price examined in the modeling study [62]. Seattle’s 1.75-cent-per-ounce SSB tax went into effect on 1/1/2018, and the SSB tax was associated with lower increases in BMI among adults in 2023 relative to people in the comparison area [63, 64]. In a recent simulation study, 2-cent-per-ounce SSB tax in California was projected to prevent 266,000 obesity cases over 10 years [65].

More recent studies have also looked at the possibility of substitution effects toward milk and juice [53]. Fletcher et al. found that children and adolescents increased their consumption of milk and juice after SSB tax implementation, and unexpectedly their caloric intake and weight did not change after the local SSB tax implementation. [54, 55, 66] Therefore, while the previous study focused on children and adolescents, it is possible that adults also turn to the substitute drinks [54, 55, 66]. In fact, Fletcher et al. found that one percentage point increase in the SSB tax drink increased caloric intake from non-SSB beverages by 7.5 cal. [54] Furthermore, in Berkeley, sales of water, untaxed fruit, vegetables, and tea drinks, plain milk all increased one year following the SSB tax [15]. The increase in caloric intake of untaxed beverages was mainly from milk and dairy-based beverages such as smoothies and milkshakes while neither the juice nor diet soda intake increased [15].

### Limitations

Our study has several limitations. First, there exists the potential for spillover effects of the tax to nearby areas. Spillover can happen when individuals who live outside the SSB tax cities shop in SSB tax cities and thus be exposed to the tax. Therefore, to reduce the likelihood of spillover effect, we limited control cities to those that were beyond 12 miles from SSB tax cities as a previous study showed that people are willing to travel and make purchases within 12 miles when the price is less in neighboring cities [31]. Second, our study makes the common shock assumption, that is, we assumed that other events which occurred at the time of or after the policy would affect SSB tax cities and control cities equally. This assumption was violated in some cities, such as San Francisco. In fact, San Francisco passed a law requiring SSB warnings on labels, advertisements, and at the point-of-sale in 2015 but was repealed later in 2021 [67]. San Francisco also excluded beverages with more than 10% of calories from added sweeteners to be sold with a toy/incentive item since 2011 [68]. In 2015, the University of California at San Francisco (UCSF) implemented a comprehensive workplace sales ban that eliminated SSB sales in all UCSF venues including cafeterias, vending machines, hospital food services, and retail outlets [69]. These policies aiming to reduce SSB consumption in San Francisco could lead to an overestimation of the effect of the tax on obesity prevalence, as San Franciscans may be decreasing consumption of SSBs due to other policies occurring during the same time frame. On the other hand, starting 2010, some California cities without soda tax started to implement restaurant kids’ meal policies to change default beverages to healthier options instead of sugary drinks [68]. Los Angeles county also required healthier beverage options including water and 100% fruit juice in county facilities since 2011 [70]. These policies aiming to reduce soda consumption in control cities may underestimate the impact of the SSB tax, as people in control cities are reducing consumption of SSBs without being exposed to the SSB tax [71]. Also, it is possible that after Berkeley implemented the SSB tax (11/2014) this could have also influenced the passing of the policies in the other treated cities: Albany, Oakland, San Francisco which passed theirs two years later (11/2016). In other words, the timing and the location of the policy are not random likely due to the presence of unmeasured confounding. To mitigate the potential for this potential source of bias, we adjusted for a large set of potential confounding variables. The near overlapping of SSB tax cities and synthetic control (Fig. 1) in the pre-SSB tax period gives us confidence that there was limited erroneous model extrapolations [34]. Third, our estimates lacked precision, and this lack of precision stems from the small sample size related to the shorter available pre- treatment periods, underpowering our analyses. We could not achieve greater balance for baseline differences between SSB tax cities and control cities either because the available data points were limited to 10 years in our study. Future studies should continue exploring the potential impact of the policy over longer periods of time. Fourth, balance between treated cities and their synthetic control counterparts was not good for some variables in certain cities (e.g. Oakland). This was also mainly due to the short available pre-treatment period data which limited our ability to achieve perfect covariate balance. However, as can be seen in Fig. 1, the pre-treatment fit was approximately okay for some cities like Oakland despite not having complete balance across some variables in Oakland and the synthetic control. Lastly, we used city-level data rather than individual-level data, the response of consumers to SSB tax depends on factors such as sex, race/ethnicity, education and income [72, 73]. Therefore, ecologic fallacy is possible here [74].

## Conclusions

Our findings were highly imprecise, and they suggested that the SSB tax could lead to a decrease or increase in obesity prevalence in California.

## Supplementary Information

Supplementary Material 1.

## Data Availability

The city-level model-based obesity prevalence data are available for purchase from the California Health Interview Survey, Neighborhood Edition, AskCHIS NE (https://askchisne.ucla.edu); the city-level socio-demographic data are freely available from the American Community Survey, ACS (https://data.census.gov). The analysis codes for implementing the ASCM are available in Supplement Text 5.

## Abbreviations

ACS: American Community Survey
ASCM: Augmented synthetic control method
ATT: Average treatment effect among the treated
BMI: Body Mass Index
CHIS: California Health Interview Survey
NE: Neighborhood Edition
SSBs: Sugar sweetened beverages
UCLA: University of California, Los Angeles
UCSF: University of California at San Francisco
US: United States
