# Supplementary material for: An application of the augmented synthetic control method within a target trial framework: the case of the soda tax policy in California
Source: BMC Public Health. 2025 Apr 11;25:1368. doi: 10.1186/s12889-025-22526-5 (PMC11987390; doi:10.1186/s12889-025-22526-5)
Supplement: Supplementary file 1 — Supplementary Material 1. [file 12889_2025_22526_MOESM1_ESM.docx]

Supplemental materials

***An application of the augmented synthetic control method within a target trial framework: the case of the soda tax policy in California***

Fan Zhao, Risha Gidwani, May Wang, Liwei Chen, Roch Nianogo

**Table of Contents**

[**Supplement Figure 1** Soda tax implementation timeline in four Californian cities. 3](#_Toc179121753)

[**Supplement Table 1** Proposed specification of a target trial to emulate via a group-level longitudinal study. 4](#_Toc179121754)

[**Supplement Text 1** Small area estimation of obesity prevalence estimates in the CHIS neighborhood edition 7](#_Toc179121755)

[**Supplement Text 2** Speficication of augmented synthetic control method (ASCM). 8](#_Toc179121756)

[**Supplement Text 3** Speficication of imputation procedures 8](#_Toc179121757)

[**Supplement Figure 4** Analytical data structure of the soda tax cities and eligible control cities. There were 121 control cities included. 10](#_Toc179121758)

[**Supplement Text 4** Eligible control cities 11](#_Toc179121759)

[**Supplement Table 2** Obesity prevalence in the pre- and post-policy periods for treated cities and their synthetic controls. 11](#_Toc179121760)

[**Supplement Table 3** Sensitivity analysis of estimated obesity prevalence difference in percentage points after implementation of the soda tax, considering one year wash-out period after SSB tax pass-through. 11](#_Toc179121761)

[**Supplement Table 4** Predicted mean squared errors (PMSE) of different model specification. 12](#_Toc179121762)

[**Supplement Figure 5** Obesity prevalence among people who were 18 years and older from 2012 to 2020 in treated cities (i.e., implemented the tax policy) and their corresponding synthetic controls. 12](#_Toc179121763)

[**Supplement Text 5** Annotated R code for the data analysis. 13](#_Toc179121764)

[**References** 14](#_Toc179121765)


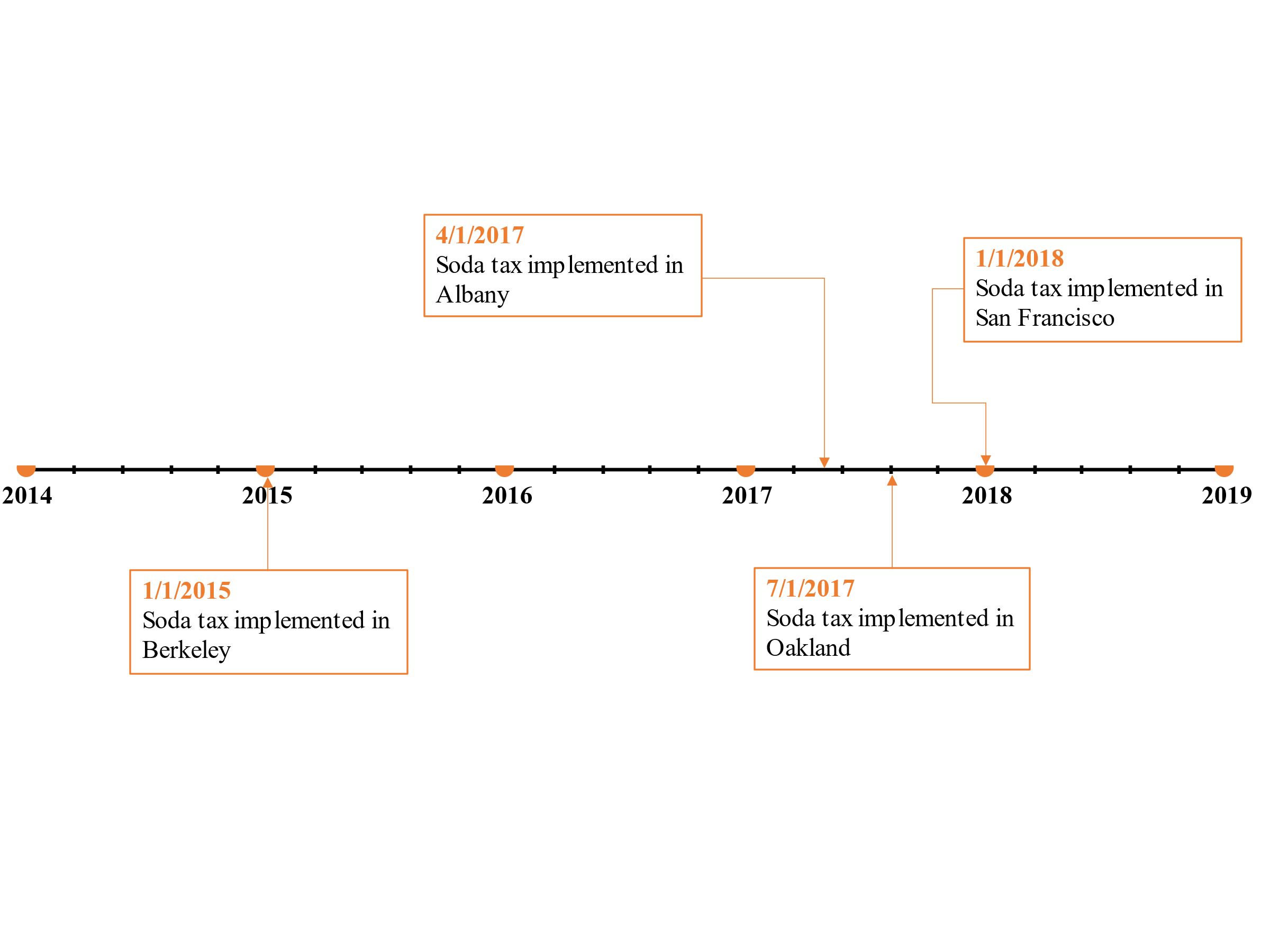


**Supplement Figure 1** Soda tax implementation timeline in four Californian cities.

**Supplement Table 1** Proposed specification of a target trial to emulate via a group-level longitudinal study.

|  | **Hypothetical cluster randomized trial** | **Emulated trial using a longitudinal panel study design at the city level** |
| --- | --- | --- |
| **Aim** | To estimate the effect of a soda tax policy on obesity prevalence in California at the city level | To estimate the effect of the soda tax policy on obesity prevalence in Berkeley, Albany, Oakland and San Francisco |
| **Eligibility** | Cities in California | Cities in California that have implemented the soda tax policy (Berkeley, Albany, Oakland and San Francisco) as the treated cities and cities in California that are more than 12 miles away from the treated cities as potential controls. |
| **Treatment strategy** | City-level 1-cent-per-ounce soda tax. Taxed SSBs include soda, sports drinks, energy drinks, and sweetened ice teas but exclude milk-based beverages, meal replacement drink, diet sodas, fruit juice, and alcohol ^1^. | City-level 1-cent-per-ounce soda tax. Taxed SSBs include soda, sports drinks, energy drinks, and sweetened ice teas but exclude milk-based beverages, meal replacement drink, diet sodas, fruit juice, and alcohol ^1^. |
| **Treatment assignment** | Cluster randomized assignment at the city level to intervention (i.e., soda tax policy) or control (i.e., will continue with status quo) | Non-randomized quasi-experimental design using longitudinal panel data. More specifically, the timing and location of the soda tax policy in each of the cities can be considered quasi-random conditional on calendar time. In addition, time-varying confounders would need to be adjusted for.  The soda tax took effect on 1/1/2015 in Berkeley, 4/1/2017 in Albany, 7/1/2017 in Oakland and 1/1/2018 in San Francisco ^2–4^. These are time zero, that is, the time at which the cities would be randomized to the policy. We will align the cities and time based on time since the soda tax policy has been enacted. |
| **Follow-up/**  **Time since randomization** | All cities (assigned to intervention or control) will be followed for 3 years post-adoption | All cities including the treated cities and control cities should have data three years from the adoption of the policy. In fact, both treated and control cities will be followed for 3 years (since this is the smallest number of years an individual city has been followed, e.g. San Francisco in our case; this will ensure the results are comparable across treated cities). In addition, longer pre-treatment periods are important for predicting synthetic controls; in other words, the more pre-treatment periods are available the better the prediction. As such, we included all available pre-treatment periods. |
| **Outcome** | Obesity prevalence at the city level | Same, obesity prevalence at the city level |
| **Causal contrast** | Intention-to-treat effect obtained by contrasting the obesity prevalence in cities assigned to the intervention group to those assigned to the control group. The results can then be aggregated across the city-level ATTs based on group. | Intention-to-treat effect and more specifically the average treatment effect on the treated (ATT) can be obtained by contrasting the obesity prevalence in cities that have implemented the soda tax to that in the corresponding synthetic control. The synthetic control is a weighted average of controls. Weights are constructed to minimize the pre-intervention difference between the treated and synthetic control. The results can then be aggregated across the city-level ATTs based on group. |
| **Statistical analysis** | Intent-to-treat effect | Intent-to-treat effect can be estimated via the augmented synthetic control method. This method creates the weights necessary to generate the synthetic controls. Adjusting for city confounders associated with soda tax implementation and obesity outcome is also necessary. |


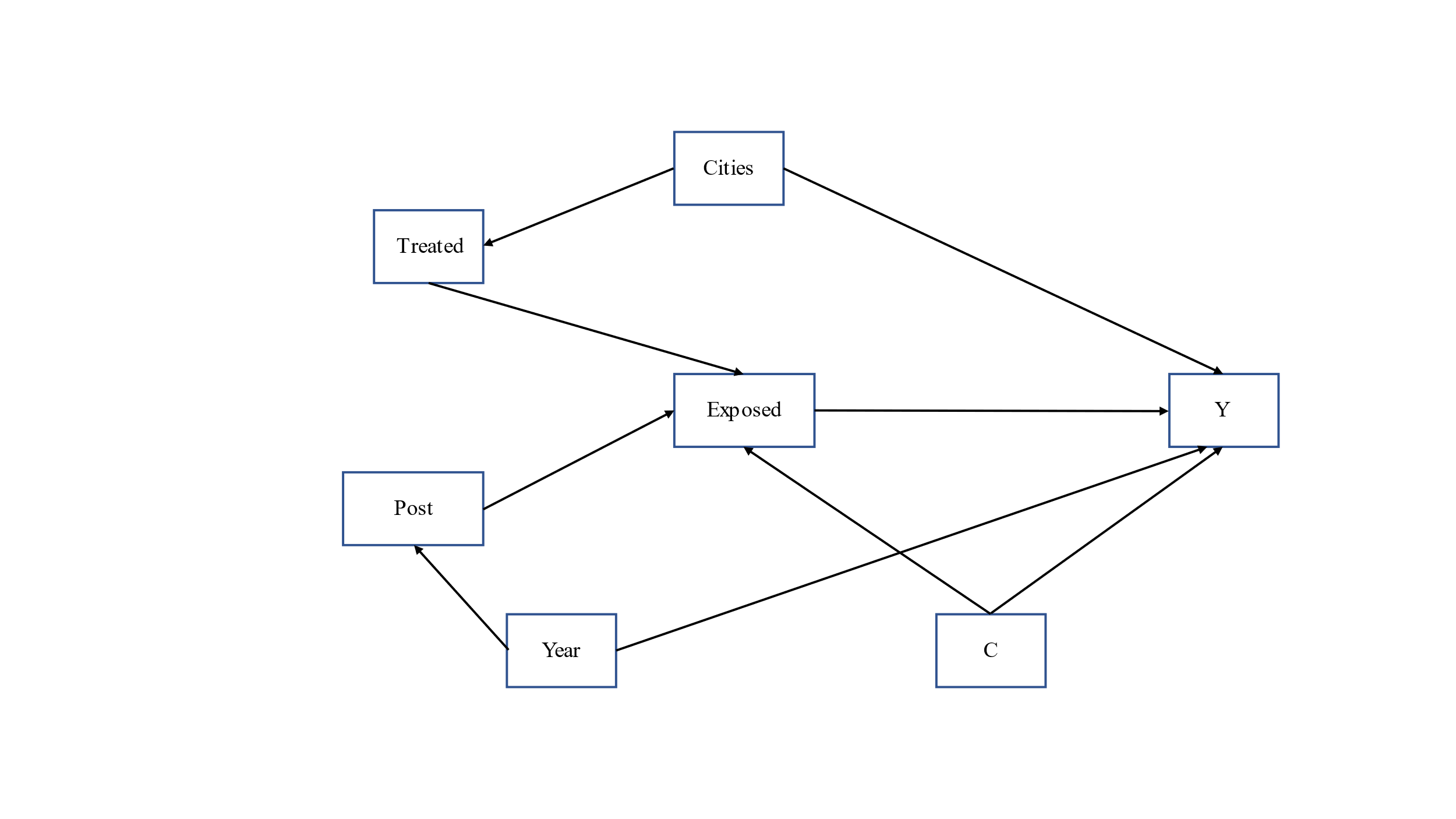


**Supplement Figure 2** Causal diagram illustrating causal structures under investigation. Treated was tax status (soda tax cities=1 vs control cities=0), Post was time (pre=0 vs post=1), Exposed was Post * Treated and Y was the outcome obesity prevalence. Confounders (C) included sex (% who were males), age (% who were 18 years and older), employment status (% who were unemployed), education (% who had bachelor’s degree or higher), race/ethnicity (% who were non-White), marital status (% who were now married), poverty (% who were below poverty), household median income (log-transformed for normality), population size (log-transformed for normality), and percentage of people who took public transportation to work.

**Supplement Text 1** Small area estimation of obesity prevalence estimates in the CHIS neighborhood edition

The California Health Interview Survey (CHIS) is a multistage cross-sectional survey of California households conducted every year since 2011 by the University of California, Los Angeles (UCLA) Center for Health Policy Research.^5^ Its neighborhood edition, CHIS NE, was obtained by Small Area Estimation (SAE) of the CHIS data which provided city-level model-based estimates of key health behaviors and conditions including city-level obesity prevalence.

The SAE model began with an individual-level generalized linear mixed model that captures individual effects as well as the survey design by including individual level fixed predictors and a random effect at the survey strata level. Then, a non-parametric function of census tract level variables was added to reflect the non-linear association between socio-demographic variables and obesity prevalence. The estimated model parameters were then applied to obtain the predicted probabilities at the individual level. Finally, individual-level predicted values were aggregated into city level estimates.^6^ The estimates were calibrated and validated against the observed values at the stratum level or external information.^6^ Included cities had populations of at least 1,000.^6^

**Supplement Text 2** Speficication of augmented synthetic control method (ASCM).

Augmented synthetic control method (ASCM) improves on the traditional synthetic control method (SCM) by using a ridge regression as the outcome model and allows negative weights on some control units.^7^

The idea of SCM is to construct a weighted average of control units that matches the treated unit’s pre-treatment outcomes. The estimated impact is then the difference in post-treatment outcomes between the treated unit and the synthetic control.^8,9^ To understand this, let j=1 be the treated unit, j=2, 3,…J be the control units. Units are observed at t=1, 2, …T. There are T_0_ number of preintervention periods, T_1_ number of postintervention periods and T_0_+T_1_=T. Unit 1 is exposed to the intervention of interest during periods T_0_+1, T_0_+2, …T. Let X_1_ be a (k×1) vector containing the values of the preintervention characteristics of the treated unit and let X_0_ be the k × J matrix collecting the values of the same variables for potential units. SCM aims to match X as closely as possible between the two groups. W is the weight that minimizes the difference between the preintervention characteristics of the treated unit, and a synthetic control (X_1_ − X_0_W).

Let Y_jt_ be the outcome of unit j at time t. Let Y_1_=(Y_1T0+1_, Y_1T0+2_,......, Y_1T_)' be the (T_1_×1) vector collecting the postintervention values of the outcome for the treated unit. Similarly, let Y_0_ be a (T_1_×J) matrix, where column j contains the postintervention values of the outcome for unit j.

The synthetic control estimator of Y_1_−Y_0_W, the comparison of postintervention outcomes between the treated unit and the synthetic control following the intervention, is interpreted as the treatment effect.^7,8^ This measures the average treatment effect among the treated (ATT). More practically and as it relates to the current study, the ATT, for instance is the contrast of the post-tax obesity prevalence in Albany to that in Albany had Albany not passed the soda tax. The phrase “had Albany not passed the soda tax” refers to a counterfactual quantity that is obtained via a weighting of the other control cities.

The augmented SCM (ASCM) uses a ridge regression outcome model to estimate the bias due to imperfect pretreatment fit, and then uses this to de-bias the SCM estimate. Ridge ASCM allows negative weights, using extrapolation to improve pre-treatment fit. The regularization parameter in ridge ASCM minimizes extrapolation at the same time by penalizing the distance from SCM weights.^7^

**Supplement Text 3** Speficication of imputation procedures

The imputeTS package in the R software specializes on time series imputation.^10^ The algorithm employs time dependencies of variables to impute the time-series data.^11^ In Moritz’s experiments, the imputeTS algorithms performed better or at least equal to all other algorithms when dealing with time series data.^11^ We specified the *na_interpolation()* function to impute missing values via linear interpolation.


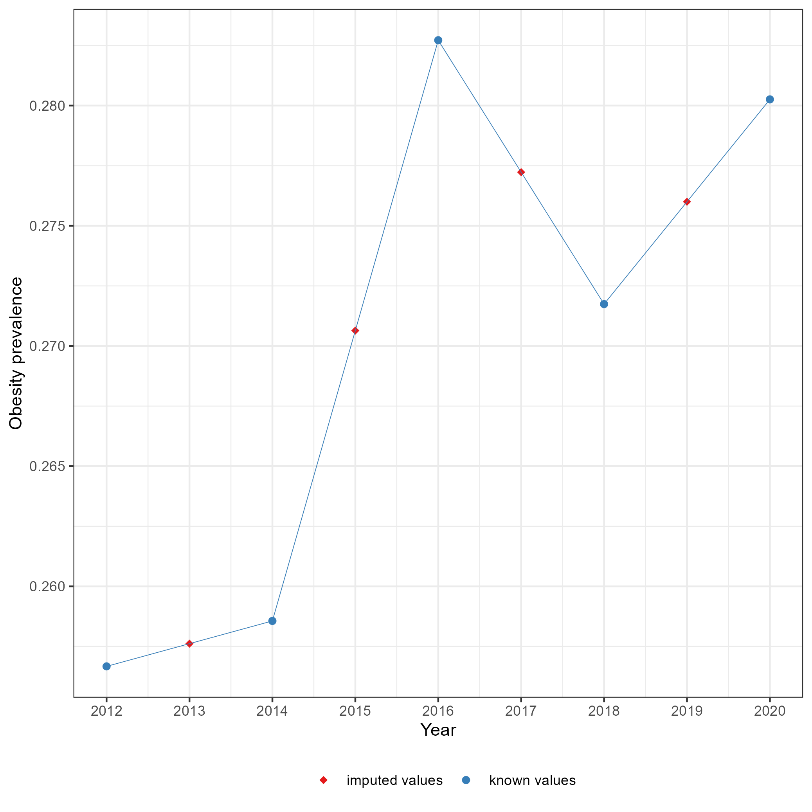


**Supplement Figure 3** Plot showing observed and imputed obesity prevalence values. City-level obesity prevalence data were only available in 2012, 2014, 2016, 2018 and 2020. Missing values were imputed for odd years using univariate time series imputation (i.e. interpolation) that employs time dependencies for other years. Blue are the known values and red are imputed values.


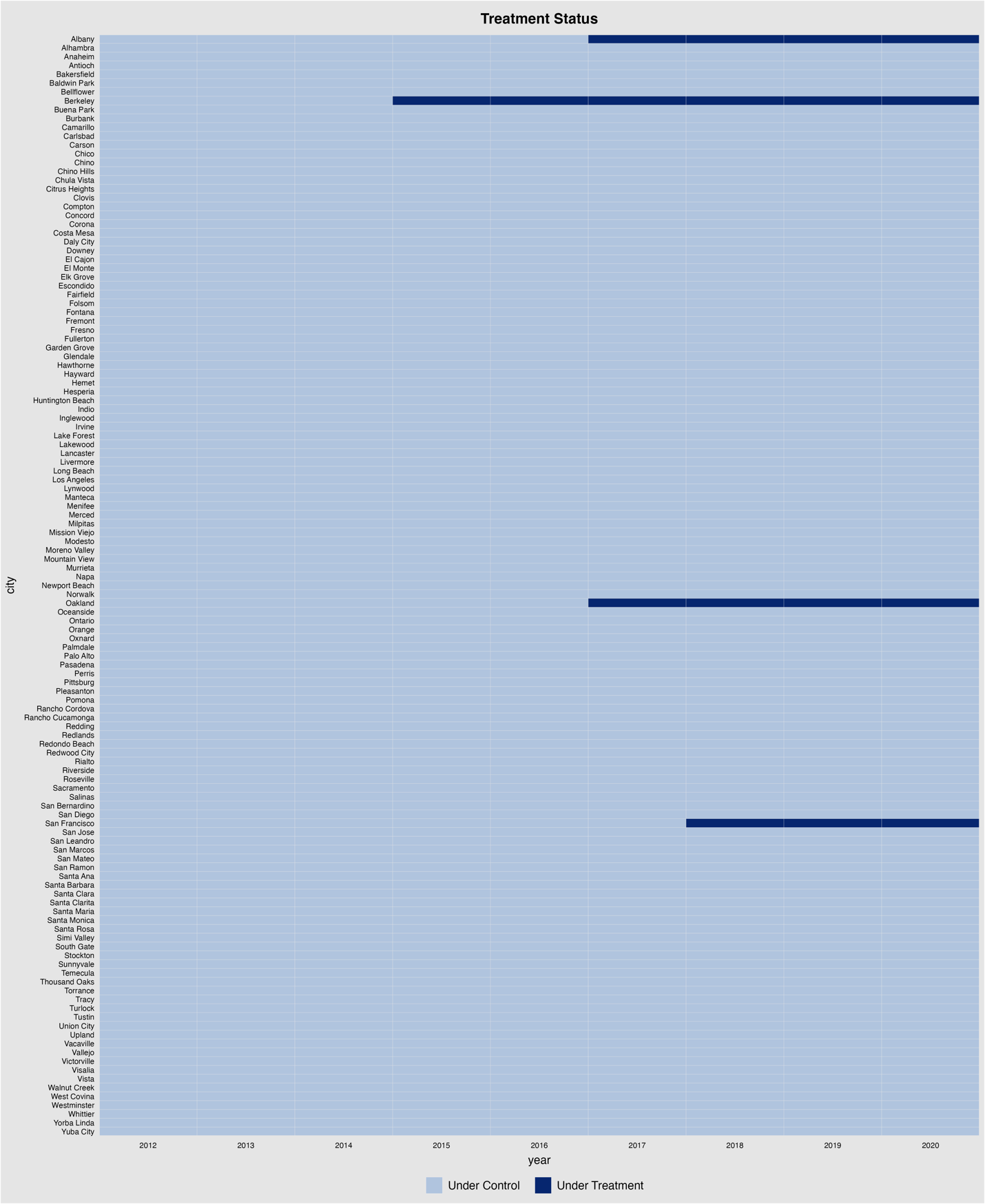


**Supplement Figure 4** Analytical data structure of the soda tax cities and eligible control cities. There were 121 control cities included.

**Supplement Text 4** Eligible control cities

There were 121 control cities that were included in our study. These were California cities that were 12 miles away from the treated cities. They include: Alhambra, Anaheim, Antioch, Bakersfield, Baldwin Park, Bellflower, Buena Park, Burbank, Camarillo, Carlsbad, Carson, Chico, Chino, Chino Hills, Chula Vista, Citrus Heights, Clovis, Compton, Concord, Corona, Costa Mesa, Daly City, Downey, El Cajon, El Monte, Elk Grove, Escondido, Fairfield, Folsom, Fontana, Fremont, Fresno, Fullerton, Garden Grove, Glendale, Hawthorne, Hayward, Hemet, Hesperia, Huntington Beach, Indio, Inglewood, Irvine, Lake Forest, Lakewood, Lancaster, Livermore, Long Beach, Los Angeles, Lynwood, Manteca, Menifee, Merced, Milpitas, Mission Viejo, Modesto, Moreno Valley, Mountain View, Murrieta, Napa, Newport Beach, Norwalk, Oceanside, Ontario, Orange, Oxnard, Palmdale, Palo Alto, Pasadena, Perris, Pittsburg, Pleasanton, Pomona, Rancho Cordova, Rancho Cucamonga, Redding, Redlands, Redondo Beach, Redwood City, Rialto, Riverside, Roseville, Sacramento, Salinas, San Bernardino, San Diego, San Jose, San Leandro, San Marcos, San Mateo, San Ramon, Santa Ana, Santa Barbara, Santa Clara, Santa Clarita, Santa Maria, Santa Monica, Santa Rosa, Simi Valley, South Gate, Stockton, Sunnyvale, Temecula, Thousand Oaks, Torrance, Tracy, Turlock, Tustin, Union City, Upland, Vacaville, Vallejo, Victorville, Visalia, Vista, Walnut Creek, West Covina, Westminster, Whittier, Yorba Linda, Yuba City.

**Supplement Table 2** Obesity prevalence in the pre- and post-policy periods for treated cities and their synthetic controls.

| **Cities and their synthetic controls** | **Mean in the pre-soda tax years** | **Mean in the 3 years after soda tax** |
| --- | --- | --- |
| Berkeley | 14.4 | 14.6 |
| Synthetic Berkeley | 16.3 | 18.9 |
| Albany | 17.3 | 17.4 |
| Synthetic Albany | 17.4 | 18.7 |
| Oakland | 24.1 | 25.3 |
| Synthetic Oakland | 17.4 | 18.7 |
| San Francisco | 13.9 | 16.4 |
| Synthetic San Francisco | 17.8 | 18.4 |

**Supplement Table 3** Sensitivity analysis of estimated obesity prevalence difference in percentage points after implementation of the soda tax, considering one year wash-out period after SSB tax pass-through.

| City, year of policy pass-through | one year wash-out period |
| --- | --- |
| Albany, 2016 | -0.0(-3.6, 3.6) |
| Berkeley, 2014 | -4.0(-25.3, 15.1) |
| Oakland, 2016 | 1.4(-6.0, 9.0) |
| San Francisco, 2016 | 3.1(-13.3, 19.9) |
| Overall | 0.1(-2.3, 2.5) |

Model adjusted for age, sex, employment status, race/ethnicity, education, median log(income), marital status, log(population) size, and percentage of people taking public transportation to work. Mean and 95%CI for all estimates.

**Supplement Table 4** Predicted mean squared errors (PMSE) of different model specification.

| Models | PMSE |
| --- | --- |
| Main ASCM model | 1.47 |
| No adjustment for income and city population | 1.47 |
| No adjustment for any covariates | 1.47 |
| SCM model | 1.47 |
| Main model adjusted for age, sex, employment status, race/ethnicity, education, median log(income), marital status, log(population) size, and percentage of people taking public transportation to work. Mean and 95%CI for all estimates. | |


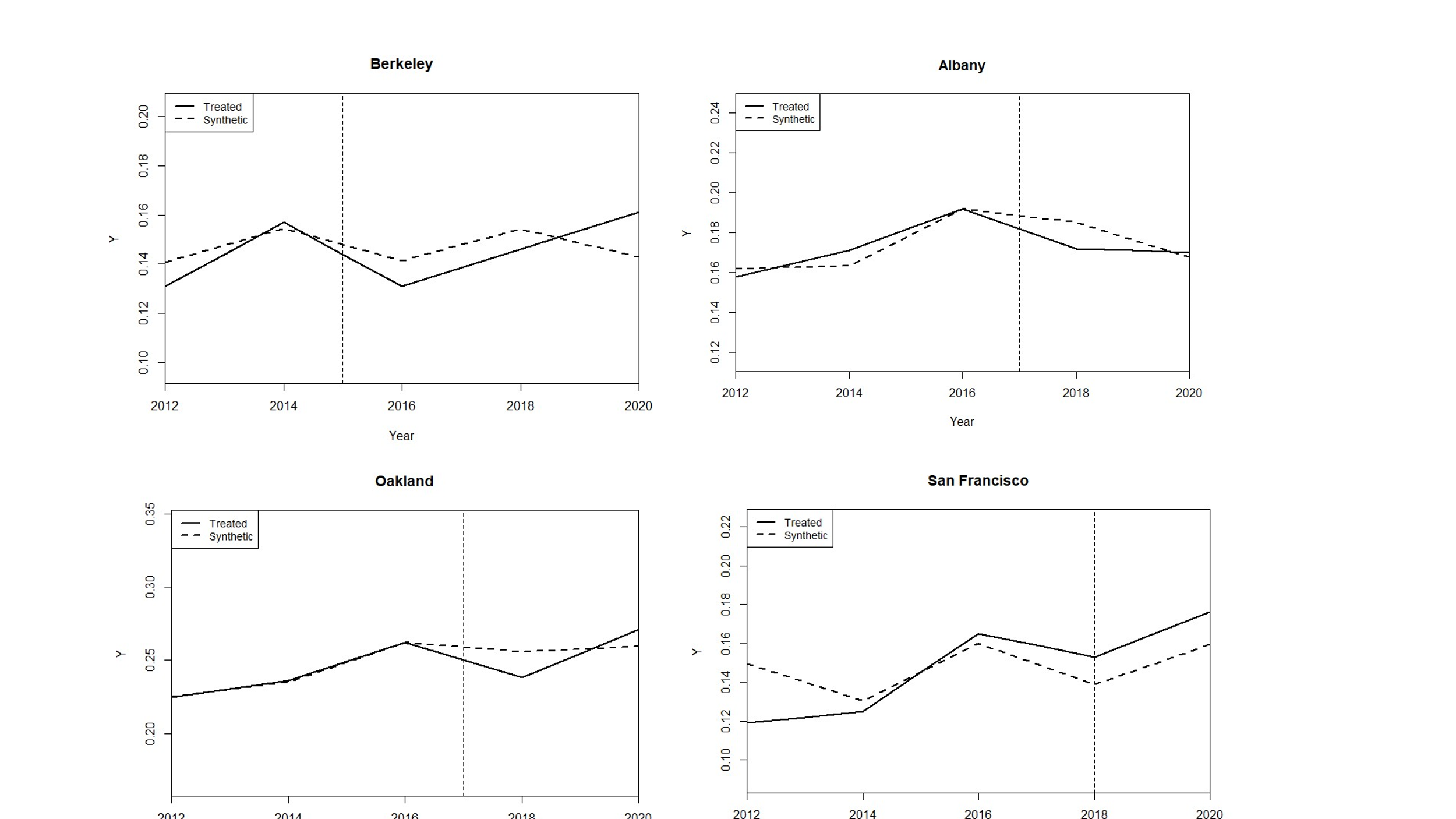


# **Supplement Figure 5** Obesity prevalence among people who were 18 years and older from 2012 to 2020 in treated cities (i.e., implemented the tax policy) and their corresponding synthetic controls when using synthetic control method (i.e., counterfactual, average obesity prevalence in treated cities had they not been treated). Dashed vertical lines represented the year the soda tax was implemented. The soda tax was implemented on 1/1/2015 in Berkeley, 4/1/2017 in Albany, 7/1/2017 in Oakland, and 1/1/2018 in San Francisco. Solid lines represented obesity prevalence in SSB tax cities, and dashed lines represented obesity prevalence in synthetic control cities.

**Supplement Text 5** Annotated R code for the data analysis.

| #------------------------------------Loading of packages------------------------   if (!require("pacman")){   install.packages("pacman", repos = 'http://cran.us.r-project.org') }   p_load("here", #for directory management: here()     "rio") #for importing data: import()   #------------------------------------Loading the data--------------------------- soda_tax_obesity <- import(here("data", "output_data",                          "soda_tax_obesity_target_trial.rds"))   #------------------------Augmented synthetic control--------------------------------- #Table 2 Estimated proportion difference after implementation of the soda tax #augsynth() for running staggered analysis p_load("augsynth")   augsynth.scm <-augsynth(form = y ~ treatedpost \| education + #formula for model: outcome ~                        male + age18 + marital + poverty + #treatment \|covariates                       transportation + unemployment +                       nonwhite + log_pop + log_income ,                     unit = city,        #Name of unit column                     time = year,        #Name of time column                     data = soda_tax_obesity,#Panel data as dataframe                     fixedeff = TRUE,    #Whether to include a unit fixed effect, default TRUE                     time_cohort = F,     #Whether to average synthetic controls into time cohorts, default FALSE                     scm = T)             #Whether to include a unit fixed effect, default TRUE   res <- summary(augsynth.scm)   res$att |
| --- |

**References**

1. Berkeley’s Tax Ordinance. City of Berkeley. Accessed April 12, 2021. http://www.healthyberkeley.com/about-berkeleys-tax-ordinance

2. City of Albany, CA. Sugar Sweetened Beverage Tax. Accessed April 12, 2021. https://www.albanyca.org/departments/finance/sugar-sweetened-beverage-tax

3. Oakland SSB Tax Requirements.pdf. Accessed April 12, 2021. http://revds.com/taxpayerpdfs/CA-GA-LA-KY-TX-forms/Oakland%20Business%20Forms/Oakland%20SSB%20Tax%20Requirements.pdf

4. Office of the Mayor. Mayor London Breed Announces Soda Tax Funding Will Provide Emergency Food for People Affected by COVID-19. Accessed April 12, 2021. https://sfmayor.org/article/mayor-london-breed-announces-soda-tax-funding-will-provide-emergency-food-people-affected

5. UCLA Center for Health Policy Research. Get CHIS Data. Accessed April 30, 2021. https://healthpolicy.ucla.edu/chis/data/Pages/confidential.aspx

6. AskCHIS Neighborhood Edition - Local Level Health Data. Accessed May 24, 2023. https://askchisne.ucla.edu/ask/SitePages/Login.aspx?ReturnUrl=%2fask%2f_layouts%2fAuthenticate.aspx%3fSource%3d%252Fask%252F%255Flayouts%252Fne%252Fdashboard%252Easpx&Source=%2Fask%2F%5Flayouts%2Fne%2Fdashboard%2Easpx

7. Ben-Michael E, Feller A, Rothstein J. The augmented synthetic control method. Published online June 2021. doi:10.3386/w28885

8. Abadie A, Diamond A, Hainmueller J. Comparative Politics and the Synthetic Control Method. *American Journal of Political Science*. 2015;59(2):495-510. doi:10.1111/ajps.12116

9. Abadie A, Diamond A, Hainmueller J. Synthetic Control Methods for Comparative Case Studies: Estimating the Effect of California’s Tobacco Control Program. Published online 2010. doi:10.1198/jasa.2009.ap08746

10. Moritz S, Gatscha S, Wang E, Hause R. imputeTS: Time Series Missing Value Imputation. Published online September 9, 2022. Accessed July 20, 2023. https://cran.r-project.org/web/packages/imputeTS/index.html

11. Moritz S. Comparison of different Methods for Univariate Time Series Imputation in R.
